# Vaccination against histomonosis limits pronounced changes of B cells and T-cell subsets in turkeys and chickens

**DOI:** 10.1016/j.vaccine.2017.06.035

**Published:** 2017-06-26

**Authors:** Taniya Mitra, Wilhelm Gerner, Fana Alem Kidane, Patricia Wernsdorf, Michael Hess, Armin Saalmüller, Dieter Liebhart

**Affiliations:** a Clinic for Poultry and Fish Medicine, Department for Farm Animals and Veterinary Public Health, University of Veterinary Medicine Vienna, Austria; b Institute of Immunology, Department of Pathobiology, University of Veterinary Medicine Vienna, Austria; c Christian Doppler Laboratory for Innovative Poultry Vaccines (IPOV), University of Veterinary Medicine Vienna, Austria

**Keywords:** *Histomonas meleagridis*, Histomonosis, Vaccination, Chicken, Turkey, Immune response

## Abstract

The protozoan parasite *Histomonas meleagridis* is the causative agent of histomonosis in gallinaceous birds. In turkeys, the disease can result in high mortality due to severe inflammation and necrosis in caecum and liver, whereas in chickens the disease is less severe. Recently, experimental vaccination was shown to protect chickens and turkeys against histomonosis but dynamics in the cellular immune response are not yet demonstrated. In the present work, different groups of birds of both species were vaccinated with attenuated, and/or infected with virulent histomonads. Flow cytometry was applied at different days post inoculation to analyse the absolute number of T-cell subsets and B cells in caecum, liver, spleen and blood, in order to monitor changes in these major lymphocyte subsets. In addition, in chicken samples total white blood cells were investigated.

Infected turkeys showed a significant decrease of T cells in the caecum within one week post infection compared to control birds, whereas vaccination showed delayed changes. The challenge of vaccinated turkeys led to a significant increase of all investigated lymphocytes in the blood already at 4 DPI, indicating an effective and fast recall response of the primed immune system.

In the caecum of chickens, changes of B cells, CD4^+^ and CD8α^+^ T cells were much less pronounced than in turkeys, however, mostly caused by virulent histomonads. Analyses of whole blood in non-vaccinated but infected chickens revealed increasing numbers of monocytes/macrophages on all sampling days, whereas a decrease of heterophils was observed directly after challenge, suggesting recruitment of this cell population to the local site of infection.

Our results showed that virulent histomonads caused more severe changes in the distribution of lymphocyte subsets in turkeys compared to chickens. Moreover, vaccination with attenuated histomonads resulted in less pronounced alterations in both species, even after challenge.

## 1 Introduction

The flagellated protozoan parasite *Histomonas meleagridis* is the aetiological agent of histomonosis (synonyms: enterohepatitis or blackhead disease) of poultry [1]. The pathogenesis can vary between species of gallinaceous birds: in turkeys (*Meleagris gallopavo*) the disease can cause high mortality whereas in chickens (*Gallus gallus*) histomonosis is generally less fatal. The pathogen primarily targets the caecum before it reaches the liver through the hepatic portal vein. The lesions are characterized by severe fibrinous inflammation of the caecum and multifocal areas of inflammation and necrosis in the liver [2]. Effective prophylactic and therapeutic options are not available for food producing birds in most industrial countries due to consumer safety regulations resulting in re-emergence of the disease and economic losses in the poultry industry [3,4].

Previous investigations on vaccination to prevent histomonosis showed that the transfer of antibodies or the use of inactivated *H. meleagridis* was not effective to protect birds from the disease [5–7]. In contrast, the application of attenuated histomonads to prevent histomonosis was earlier demonstrated [2] and recently performed experimental studies showed that clonal *in vitro* attenuated *H. meleagridis* are effective and safe in protecting turkeys and chickens [7–10]. However, data on the immune response against histomonads are limited. Varying cytokine expression profiles in caecum and liver between chickens and turkeys indicated an innate immune response of chickens against histomonosis [11]. In the same work, the occurrence of different populations of lymphocytes in liver and spleen by immunohistochemistry was demonstrated. Moreover, co-infection of *Heterakis gallinarum* and *H. meleagridis* of chickens showed the involvement of T cells in the caecum with induction of Th1 and Th2 type cytokines [12]. The activation of the local humoral immune response was demonstrated by detecting specific antibodies in different parts of the intestine of chickens infected with histomonads [13]. Anyhow, there are no data available about detailed changes in lymphocyte distribution following *H. meleagridis* infection or vaccination.

Therefore, the aim of the present work was to investigate changes in the kinetics of lymphocytes during inoculation of turkeys and chickens with attenuated and virulent *H. meleagridis*. CD4^+^ and CD8α^+^ T cells together with B cells in different organs and blood were investigated by flow cytometry (FCM) for the first time to obtain insights into the local and systemic cellular immune response after vaccination and/or infection. In addition, data on the cell dynamics of B cells, total T cells, macrophages/monocytes together with heterophils in whole blood of chickens were generated by FCM analysis following inoculation with *H. meleagridis* in chickens.

## 2 Materials and methods

### 2.1 Birds

A total of sixty turkeys (B.U.T. 6™; Aviagen Turkeys Ltd, Tatten-hall, UK) and the same number of specific pathogen free (SPF) layer type chickens (VALO, BioMedia, GmBH, Osterholz-Scharmbeck, Germany) were included in the present study. At the first day of life every bird was marked with subcutaneously fixed tags for identification.

### 2.2 Preparations of parasites for inoculation

The clonal culture *H. meleagridis*/Turkey/Austria/2922-C6/04 [14] was co-cultivated with intestinal flora of the host bird before used for vaccination and infection of the birds: attenuated histomonads, established by long-term cultivation for 295 passages, were used for vaccination whereas for infection the virulent cultured histomonads (21 passages) were administered as previously described [7]. Both cultures were stored at −150 °C prior to inoculation. 6 × 10^5^ cells of *H. meleagridis* in 600 μl culture medium consisting of Medium 199 with Earle’s salts, L-glutamine, 25 mM HEPES and L-amino acids (Gibco™ Invitrogen, Lofer, Austria), 15% foetal calf serum (FCS) (Gibco™ Invitrogen) and 0.66 mg rice starch (Sigma-Aldrich, Vienna, Austria) were administered per bird, split between the oral and cloacal route using a syringe together with a crop tube, respectively a pipette. Birds of the control groups were sham infected with the equal volume of pure culture medium.

### 2.3 Setup of the in vivo trial

Water and feed (unmedicated turkey, respectively chicken starter feed) were provided *ad libitum*, except for 5 h of feed restriction after inoculation. The different groups consisted of 15 birds of each species and were kept separated in four rooms depending on the inoculation scheme: vaccinated turkeys (VT), vaccinated chickens (VC), infected turkeys (IT), infected chickens (IC), vaccinated and infected turkeys (VIT), vaccinated and infected chickens (VIC), control turkeys (CT) and control chickens (CC) (Table 1). Vaccination of groups VIT and VIC was applied on the first day of life. The challenge infection of the same birds was performed 28 days later together with the inoculation of the IT and IC groups. On the same day (28th day of life) birds from the only vaccinated group were inoculated with the attenuated strain and control birds were inoculated with culture medium only. From this day onwards, 3 previously determined birds (ascending order of tag numbers) per group were sacrificed 4, 7, 10, 14 and 21 days post inoculation (DPI).

### 2.4 Clinical examination, post-mortem and sampling

Behaviour, plumage, faeces, feed and water intake together with body weight were examined throughout the experiment for any clinical signs indicative for histomonosis. Re-isolation of viable parasites from cloacal swabs of every bird was performed in intervals of 2–3 days following vaccination and/or infection to confirm the successful inoculation. For that, after sampling each swab was placed into a 2 ml Eppendorf tube containing culture medium as described above and incubated at 40 °C. Following a propagation period of 2–3 days the re-isolations were microscopically examined. Blood samples of every bird were collected directly before the birds were killed. Birds that were killed or had to be euthanized were anaesthetized by intravenous application of thiopental (Sandoz, Kundl, Austria) before bleeding to death. Dead birds were necropsied and pathological changes in caecum and liver were evaluated using a previously established lesion score (LS) system: LS 0 was applied for normal organs whereas LS 1–4 classified mild to severe changes [13]. Caecum, liver and spleen were weighed and collected in cold phosphate buffered saline (PBS) (Gibco™ Invitrogen) containing 2% FCS (Gibco™ Invitrogen) (PBS + FCS) as soon as possible after death and further processed as described below.

### 2.5 Cell isolation

Single cell suspensions were prepared according to standard procedures from caecum, liver, spleen and blood PBMCs together with total white blood cells. Brief descriptions are given below for each type of sample.

#### 2.5.1 Caecum

Intraepithelial lymphocytes (IELs) were isolated from both caeca of each bird as described previously [15] with some modification. In detail, faeces were removed and the organ samples were rinsed with cold PBS + FCS. After cleaning, the caeca were cut longitudinally in pieces of approximately 1 cm before isolation was performed in a solution of 50 ml PBS (Gibco™ Invitrogen) containing 500 μl of 1 M DTT (Sigma-Aldrich) and 10 μl of 0.5 M EDTA at 37 °C for 30 min during continuous stirring. The sediment consisting of sloughed tissue was left behind and the supernatant was transferred into two 50 ml tubes in equal amounts. The tubes were filled up with cold PBS + FCS and centrifuged at 4 °C, 220g for 10 min. The supernatant was collected passed through 40 μm nylon cell strainer (BD Falcon©), whereas the pellet was resuspended in 50 ml cold PBS + FCS to obtain more single cells by further centrifugation steps at 4 °C, 350g for 10 min. By that, single cells of the supernatants from the centrifugation steps were obtained before passed through the cell strainer. Following centrifugation the pellet was resuspended in 10 ml PBS + FCS.

The prepared suspension was then slowly layered above a double volume of Histopaque®-1077 (Sigma-Aldrich, Vienna, Austria) for density gradient centrifugation. The cells from interphase layer were collected and washed. Finally, the pellet was dissolved in 1 ml of the same solution.

#### 2.5.2 Liver and spleen

Single cells from liver and spleen were obtained by mechanical dissection. Isolation of lymphocytes was performed by crushing the liver (plunger of a syringe) and by tearing apart the spleen (two forceps) in petri dishes containing up to 30 ml cold PBS + FCS. The cells were then separated from the remaining tissue through a 40 μm nylon cell strainer (BD Falcon©) in a 50 ml tube. Following sedimentation of bigger tissue pieces for 10 min the supernatant was collected and centrifuged at room temperature, 350g for 10 min. The pellet was resuspended in 5 ml cold PBS + FCS and separated by density gradient as described for IELs. Mononuclear cells were finally resuspended in 5 ml cold PBS + FCS.

#### 2.5.3 Blood

For the separation of peripheral blood mononuclear cells (PBMCs) approximately 3 ml of blood was collected from the wing vein of each bird and immediately transferred into blood collection tubes containing EDTA (EDTA KE/1.3, Sarstedt, Nümbrecht, Germany). The blood was mixed with an equal volume of cold PBS + FCS and subjected to density gradient separation under the conditions mentioned above after which, the obtained PBMCs were diluted in 1 ml cold PBS + FCS.

For total white blood cells, between 0.75 and 1 ml of blood per bird was collected in EDTA tubes (EDTA KE/1.3, Sarstedt). The samples were further treated with TransFix^®^ solution (Cytomark, Buckingham, UK) according to manufacturer’s protocol for conservation of the cells. Agitation was performed using a Stuart general rotator, STR4 (Bibby Scientific Limited, Staffordshire, UK) in circular rotation with 45° angle of the tubes with 15 rpm at room temperature, to ensure a proper diffusion.

### 2.6 Flow cytometry

#### 2.6.1 FCM staining protocol

Lymphocytes of blood and tissues were microscopically examined for their viability using Trypan Blue and counted by a Neubauer hemocytometer (Sigma-Aldrich, Vienna, Austria). A concentration of 2 × 10^7^ cells/ml PBS + FCS was adjusted before the cells were stained. Different combinations of monoclonal antibodies (mAbs) were used for immunophenotyping of the isolated cells. Detailed information on antibody combinations for the two species and, where applicable, their fluorescence labelling by second-step reagents is given in Table 2. The final concentration of every antibody was determined by titration and the respective isotype controls were included.

For staining of mononuclear cells isolated from blood and organs, 20 μl of the adjusted cell suspension was transferred into wells of 96-well microtiter plates (Sarstedt, Nümbrecht, Germany) together with the respective primary antibodies, except anti-human CD3ε, for incubation for 30 min at 4 °C. Afterwards, cell pellets obtained by centrifugation at 4 °C, 250*g* for 4 min were washed two times with cold PBS + FCS. For biotinylated antibodies the secondary reagent Brilliant Violet 421™ Streptavidin (BioLegend, San Diego, CA, USA) was applied. Following another incubation step for 30 min at 4 °C further washing was performed. The cells were fixed with BD fixation buffer (BD Biosciences, San jose, CA, USA) according to manufacturer‘s protocol. Intracellular staining with the anti-human CD3ε mAb CD3-12 was performed after fixation and permeabilization. To achieve this, the BD Cytofix/Cytoperm™ fixation/permeabilization kit (BD Biosciences) was employed according to manufacturer’s instructions. Afterwards the cells were incubated with CD3-12 antibody for 30 min followed by two washing steps. Finally, the pellets were resuspended in 200 μl cold PBS + FCS and kept at 4 °C until FCM analysis.

Total white blood cells were analysed according to a previously established protocol [16] with slight modifications. Briefly, blood samples were processed in BD Trucount Tubes^®^ (BD Biosciences, Austria) and incubated with mouse anti-chicken CD45-PerCp (AbD Serotec), mouse anti-chicken Bu-1-APC (SouthernBiotech), mouse anti-chicken TCR-γδ-FITC, mouse anti-chicken CD8α-FITC, mouse anti-chicken CD4-FITC and mouse anti-chicken KUL-01-RPE (SouthernBiotech) (see Table 2 for details on antibodies) before FCM was performed.

#### 2.6.2 FCM analysis

FCM of stained cells was performed on a FACSCanto II (BD Biosciences, San Jose, CA) flow cytometer equipped with three lasers (405, 488 and 633 nm) and a high throughput sampler (HTS). At least 40,000 lymphocytes per sample were recorded. Analysis of FCM raw data was performed by FACSDiva Software version 6.1.3 (BD Biosciences).

#### 2.6.3 Determination of absolute cell counts

For calculation of absolute cell numbers, percent values obtained by lymphocyte-subgating were multiplied by the total lymphocyte counts obtained by hemocytometer. After that, every cell population was calculated per gram or per ml according to the weight or volume of the tissue or blood, respectively, taken for the extraction procedure.

For total white blood cell counts at least 10,000 Trucount^®^ beads were recorded in each sample and absolute numbers of individual cell populations were calculated using following formula: absolute cell count/μl blood = (cells counted/beads counted) × (total content of beads per tube/blood volume per tube) [16].

### 2.7 Statistical analysis

For statistical calculation of numbers of the different cell populations, the mean values of cell populations from three birds per group were used for each time point. Samples obtained from vaccinated and/or infected groups were individually compared to the equivalent cells isolated from birds of the negative control group. Significant differences were calculated using student’s T-test, with a p-value ≤0.05.

## 3 Results

### 3.1 Clinical signs and post mortem

Turkeys infected with virulent histomonads (group IT), showed first clinical signs, such as depression, diarrhoea and ruffled feathers, on 7 DPI. The severity of histomonosis increased in birds of this group and therefore the remaining birds had to be euthanized before the pre-last sampling day, i.e. 14 DPI. In contrast, no clinical signs were noticed in any other group. Re-isolations of histomonads from cloacal swabs confirmed the presence of the parasite in every group with the exception of control birds (data not shown).

**Fig. 1:**
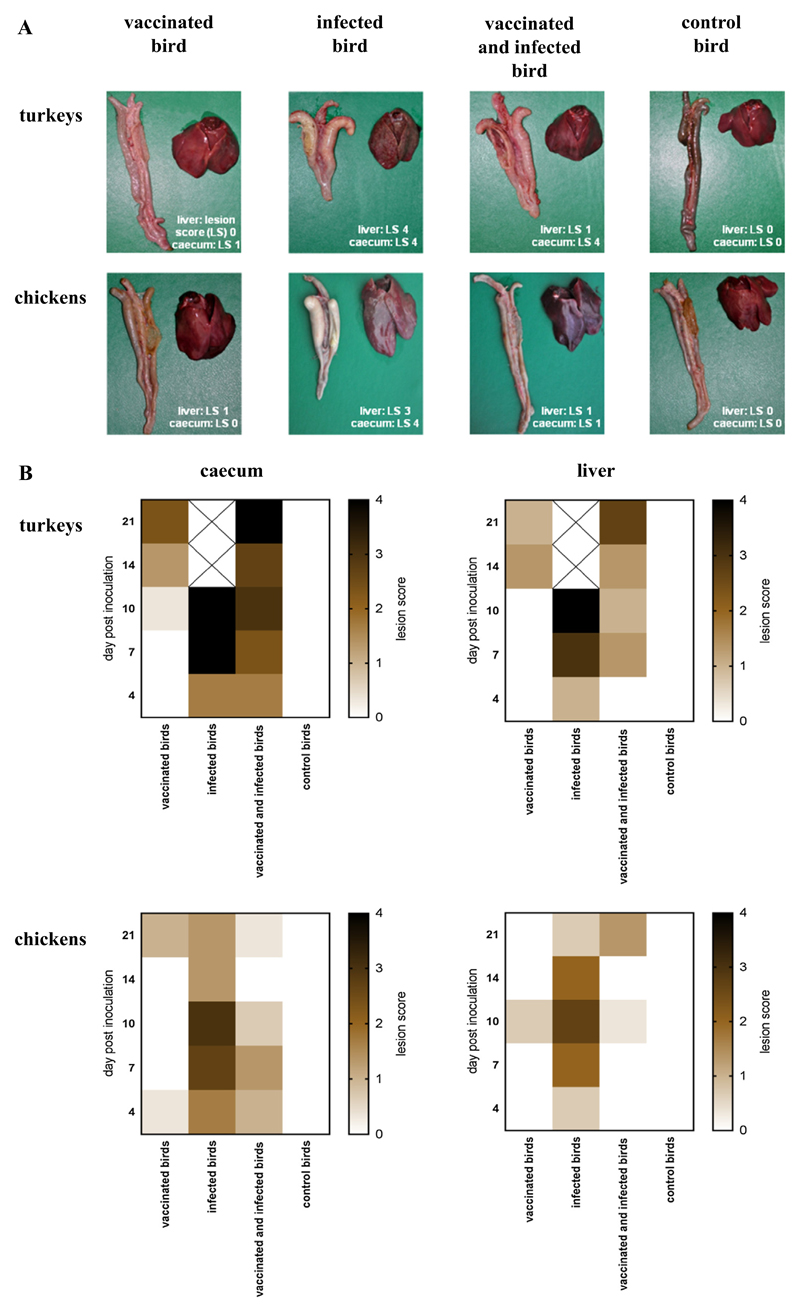
Pathological changes in caecum and liver following vaccination and/or infection of chickens and turkeys. (A) Differences in the severity of lesions using defined lesion scores (ls 0–4) between the different groups are shown exemplarily at 10 day post inoculation in caecum and liver of 1 bird of every group. (B) Heat map comparatively indicating the mean lesion score of 3 birds of every group on the different days post inoculation (Software: Graph Pad PRISM^®^ 7.02).

Different grades of pathological changes (Fig. 1A) and the mean LS on the respective sampling day of every group determined during the post mortem procedure are summarized in Fig. 1B. In the IT group first signs of inflammation were observed in caecum and liver at 4 DPI which later on increased until 10 DPI with the maximum LS 4 in both organs. In group VIT lesion scores in the caecum and liver were milder on the respective sampling days and increased until 21 DPI in the caecum to LS 4 and in the liver to LS 2.5. First lesions in only vaccinated turkeys (VT group) were observed at 10 DPI in the caecum and 4 days later in the liver. The obtained scores were rather low, between LS 1 and LS 3. The severity of lesions in chickens was graduated from LS of 3 in the caecum and 2.5 in the liver of IC at 10 DPI to lower values in group VC. Chickens of group VIC and VC entirely displayed LS below 2. None of the control birds in group CT or CC showed lesions at any sampling day.

### 3.2 Flow cytometry analysis

#### 3.2.1 Cross-reactivity of mAb CD3-12 with turkey T cells

**Fig. 2:**
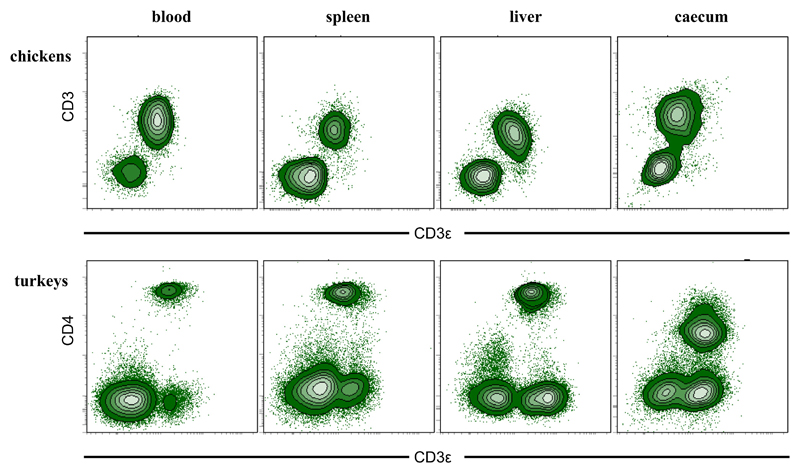
Evaluation of cross-reactivity of anti-human CD3ε antibody clone CD3-12 with lymphocytes from chickens and turkeys. Lymphocytes of caecum, liver, spleen and blood of both species were isolated and stained with the anti-human CD3ε antibody clone CD3-12 (x-axes, both species) and an anti-chicken CD3 mAb (clone CT3, y-axis, top panel) or an anti-chicken CD4 mAb (clone CT4, y-axis, bottom panel). Data is representative of experiments with cells isolated from 3 different chickens or turkeys each.

**Fig. 3:**
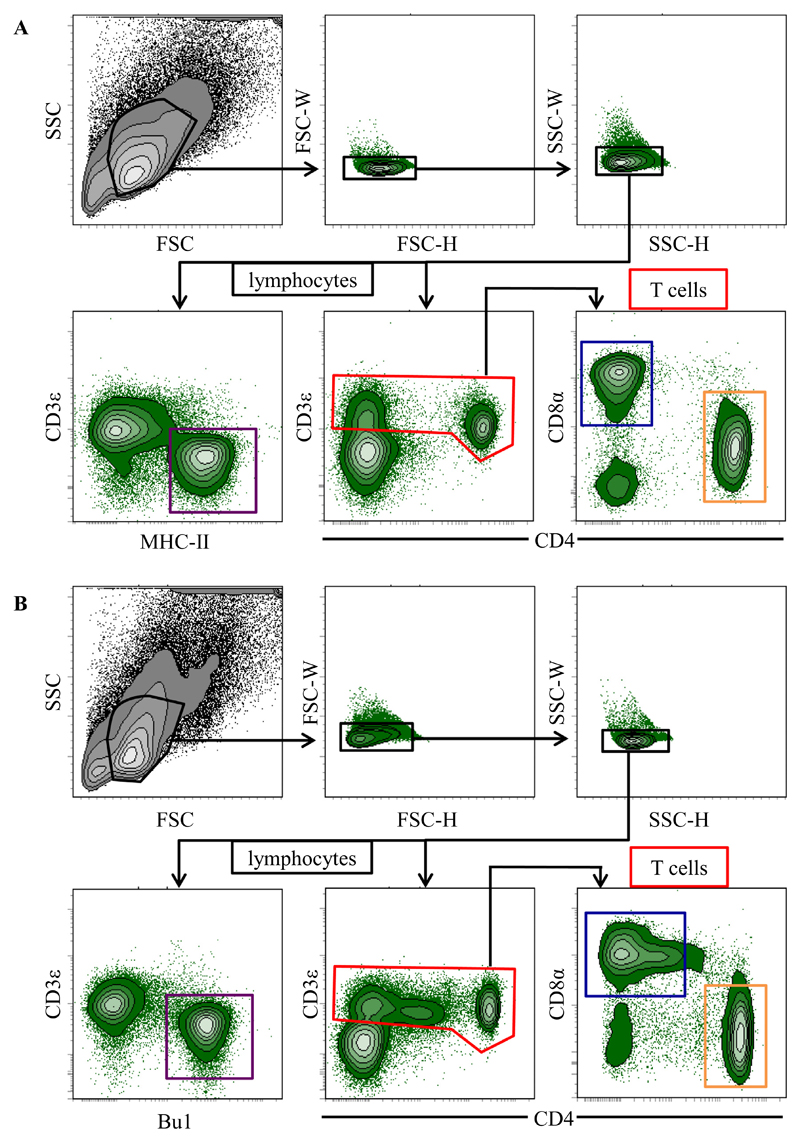
Gating strategy for lymphocytes in multicolour flow cytometry analysis. Lymphocytes isolated from (A) turkeys and (B) chickens, were gated according to their light scatter properties. Potential doublet cells were excluded by consecutive FSC-H/FSC-W and SSC-H/SSC-W double discrimination gates. Single cells were analysed for CD3 expression (middle panel) and gated as CD3ε^+^ T cells (red gate, a + b). CD3ε^+^ T cells were further analysed for CD3ε^+^CD4^+^CD8α^−^ T cells (orange gate) and CD3ε^+^CD4^−^CD8α^+^ cells (blue gate). In parallel samples CD3ε^−^MHC-II^+^ B cells (turkey, a, purple gate) or CD3ε^−^Bu1^+^ B cells (chicken, b, purple gate) were identified. The gating strategy is shown as a representative example for splenocytes isolated from control birds at 10DPI and was performed accordingly for all analysed organs. (For interpretation of the references to color in this figure legend, the reader is referred to the web version of this article.)

As no turkey-specific anti-CD3 antibody was available, cross-reactivity of the rat anti-human antibody CD3ε (clone CD3-12) was tested for lymphocytes of chicken and turkey in initial experiments. The mAb CD3-12 recognizes an epitope of 14 amino acid residues within the cytoplasmic domain of the CD3ε molecule. By using Basic Local Alignment Search Tool (BLAST^®^) the amino acid sequences comprising this epitope of the following species were compared: *Meleagris gallopavo; Gallus gallus and Homo sapiens* (Table 3). The BLAST^®^ showed that within the 14 amino acids only one amino acid, situated at N-terminus, is different for turkeys and chickens compared to the human sequence. FCM with lymphocytes isolated from chicken blood, spleen, liver, caecum and stained with rat anti-human CD3ε (clone CD3-12) versus mouse anti-chicken CD3 (clone CT3) revealed that both antibodies target the same cell population (Fig. 2, top panel). In parallel, lymphocytes derived from the same organs of turkeys were labelled also with rat anti-human CD3ε (clone CD3-12) and co-stained with a mouse anti-chicken CD4 mAb (with a documented cross-reactivity against turkey CD4 [17]). Results revealed that all CD4^+^ cells were co-stained by the CD3-12 antibody in all investigated organs, indicating that these cells represent CD3^+^CD4^+^ T cells (Fig. 2, bottom panel). Moreover, a separate population of CD3-12^+^ but CD4^−^ population of cells was obtained, representing CD3ε^+^ CD8α^+^ and γδ T cells (see also Fig. 3A, bottom panel).

#### 3.2.2 Gating strategy for identification of B cells, CD4^+^ and CD8α^+^ T cells in chickens and turkeys

FCM analyses were performed with lymphocytes isolated from caecum, liver, spleen, and blood of *H. meleagridis* infected/vaccinated turkeys and chickens. A uniform gating hierarchy was used throughout all sampling days and for both species (Fig. 3A + B). After gating on putative lymphocytes by light scatter properties, doublet cells were excluded in two consecutive gates based on uniform forward scatter and side scatter width versus height properties. For both species, total T cells were identified in a gate (illustrated in red) comprising by CD3ε^+^CD4^+^ T cells and CD3ε^+^CD4^−^ T cells and further sub-gated for analysis of CD4 and CD8α expression. Two subsets were identified in this way: CD3ε^+^CD4^+^CD8α^−^ (orange window) and CD3^+^CD4^−^CD8α^+^ T cells (blue window). For the identification of B cells different marker combinations were used for turkeys and chicken. Due to the lack of a B-cell specific antibody in turkeys, we decided to identify putative B cells in this species by a CD3ε^−^MHC-II^+^ phenotype (Fig. 3A, bottom panel, left, purple gate) since it has been shown that turkey B cells express MHC-II (clone 2G11) [18]. Chicken B cells were identified by the commercially available Bu1 (clone AV20) mAb in parallel to CD3ε staining. In this way CD3ε^−^Bu1^+^ cells were identified (Fig. 3B, bottom panel, left, purple gate) and designated as B cells for all chicken samples.

Following the identification of these lymphocyte subsets by the illustrated gates, the mean of absolute numbers of CD4^+^ (CD3ε^+^-CD4^+^CD8α^−^), CD8α^+^ (CD3ε^+^CD4^−^CD8α^+^) and B (CD3ε^−^MHC-II^+^ or CD3ε^−^Bu1^+^) cells in caecum, liver, spleen, and blood of turkeys and chickens were calculated and are comparatively given between 4 and 21 DPI.

#### 3.2.3 Caecum

**Fig. 4:**
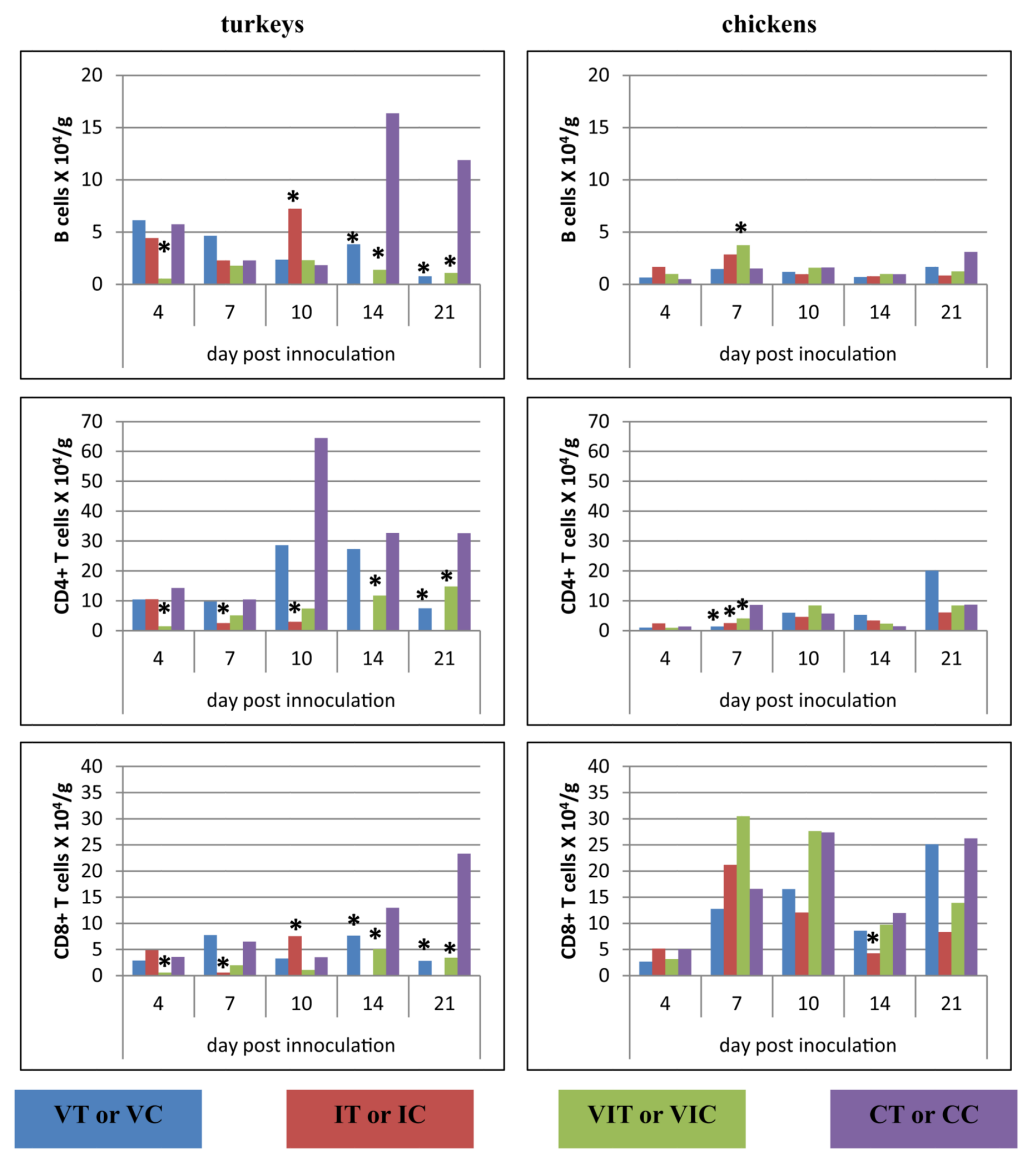
Alterations of B cells, CD4^+^ and CD8α^+^ T cells following vaccination and/or infection analysed by flow cytometry in the caecum. Absolute cell numbers of B cells (CD3ε^−^MHC-II^+^, turkey; CD3ε^−^Bu1^+^, chicken), CD4^+^ T cells (CD3ε^+^CD4^+^CD8α^−^) and CD8α^+^ T cells (CD3ε^+^CD4^−^CD8α^+^) were determined in groups of vaccinated turkeys (VT), vaccinated chickens (VC), infected turkeys (IT), infected chickens (IC), vaccinated and infected turkeys (VIT), vaccinated and infected chickens (VIC), control turkeys (CT) and control chickens (CC). Data represent the mean absolute cell number of 3 birds/group. *Significant different to control group of the respective species (P ≤ 0.05).

In the caecum, at 4 DPI, both investigated T-cell subsets (CD3ε^+^-CD4^+^CD8α^−^, CD3ε^+^CD4^−^CD8α^+^) and B cells significantly decreased in birds of group VIT in comparison to controls (Fig. 4). Three days later, a decrease of the T-cell subsets was observed in birds of group IT whereas in all inoculated chicken groups IC, VIC, VC only CD4^+^ T cells were found decreased. On the same day, B cells of chicken were only altered in group VIC, when higher numbers were counted. At 10 DPI, only turkeys of group IT were affected by a significant decrease of CD4^+^ T cells but increased numbers of CD8α^+^ T cells and B cells. On the last two sampling days, the groups VIT and VT showed a reduction of all lymphocyte subpopulations but not all values were statistically relevant. The significantly decreased number of CD8α^+^ T cells at 14 DPI of chickens of group IC was the only difference in chicken at the final stage of the experiment.

#### 3.2.4 Liver

**Fig. 5:**
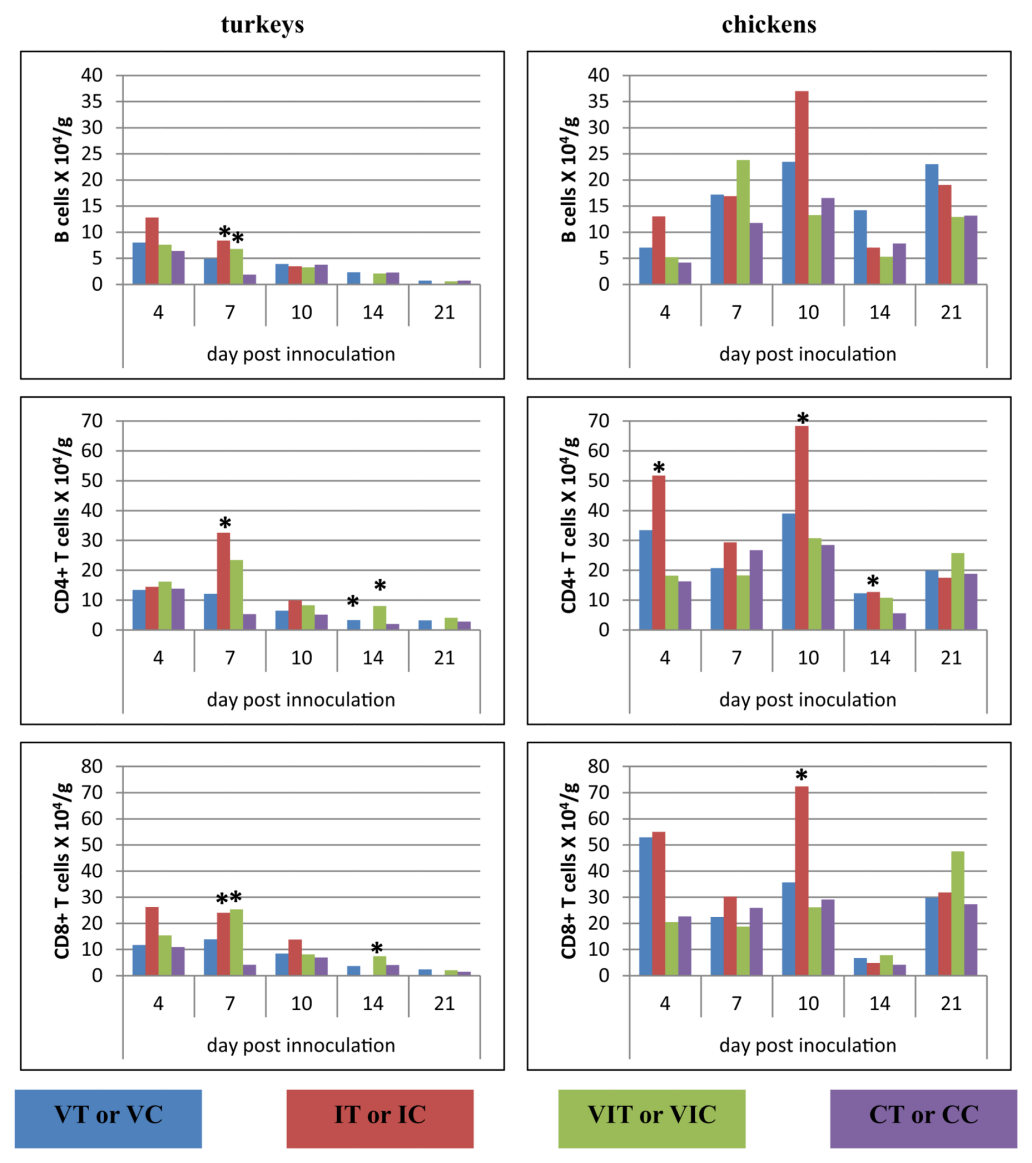
Alterations of B cells, CD4^+^ and CD8α^+^ T cells following vaccination and/or infection analysed by flow cytometry in the liver. Absolute cell numbers of B cells (CD3ε^−^MHC-II^+^, turkey; CD3ε^−^Bu1^+^, chicken), CD4^+^ T cells (CD3ε^+^CD4^+^CD8α^−^) and CD8α^+^ T cells (CD3ε^+^CD4^−^CD8α^+^) were determined in different groups of turkeys and chickens at different days post inoculation. Data represent the mean absolute cell number of 3 birds/group. *Significant different to control group of the respective species (P ≤ 0.05).

In the liver, at the early stage of infection (4 DPI), only chickens of group IC showed a significant increase of CD4^+^ T cells (Fig. 5). Later on at 7 DPI, elevations of all types of lymphocytes were observed in turkeys of all inoculated groups with significant changes in group IT compared to CT. Similar elevation were also visible for VIT. Infected chicken (IC) showed a robust increase of both T-cell subsets at 10 DPI. Both CD4^+^ and CD8α^+^ T cells significantly increased at 14 DPI in group VIT whereas only a higher amount of CD4^+^ T cells was found in the VT group. At the final sampling day the cell numbers in livers of all birds did not vary significantly from control birds.

#### 3.2.5 Spleen

**Fig. 6:**
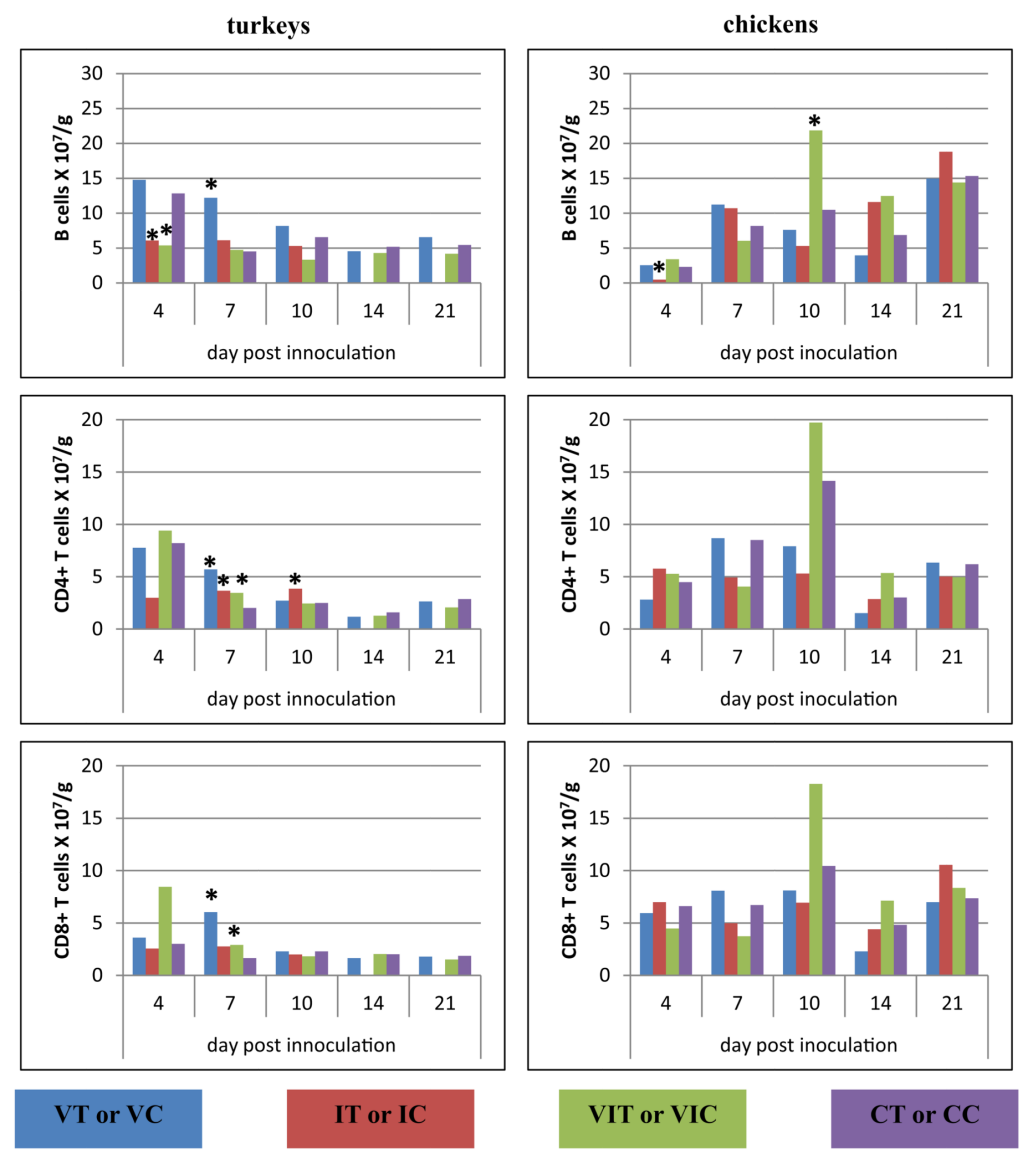
Alterations of B cells, CD4^+^ and CD8α^+^ T cells following vaccination and/or infection analysed by flow cytometry in the spleen. Absolute cell numbers of B cells (CD3ε^−^MHC-II^+^, turkey; CD3ε^−^Bu1^+^, chicken), CD4^+^ T cells (CD3ε^+^CD4^+^CD8α^−^) and CD8α^+^ T cells (CD3ε^+^CD4^−^CD8α^+^) were determined in different groups of turkeys and chickens at different days post inoculation. Data represent the mean absolute cell number of 3 birds/group. *Significant different to control group of the respective species (P ≤ 0.05).

Within lymphocytes isolated from spleen (Fig. 6), most changes were observed within one week post inoculation: at 4 DPI, B cells of groups IT, VIT and IC were significantly decreased in comparison to controls, whereas T-cell subsets did not differ significantly. At 7 DPI, turkeys of group VT showed an increase of B cells as well as CD4^+^ T cells in all inoculated groups and CD8α^+^ T cells in group VT and VIT. Lymphocytes in spleen samples collected from chickens on the same day did not differ significantly but an elevation of B cells in VIC was observed at 10 DPI. On the same day in turkeys CD4^+^ T cells were increased as the last cellular aberrance in the time course p.i.

#### 3.2.6 Blood

**Fig. 7:**
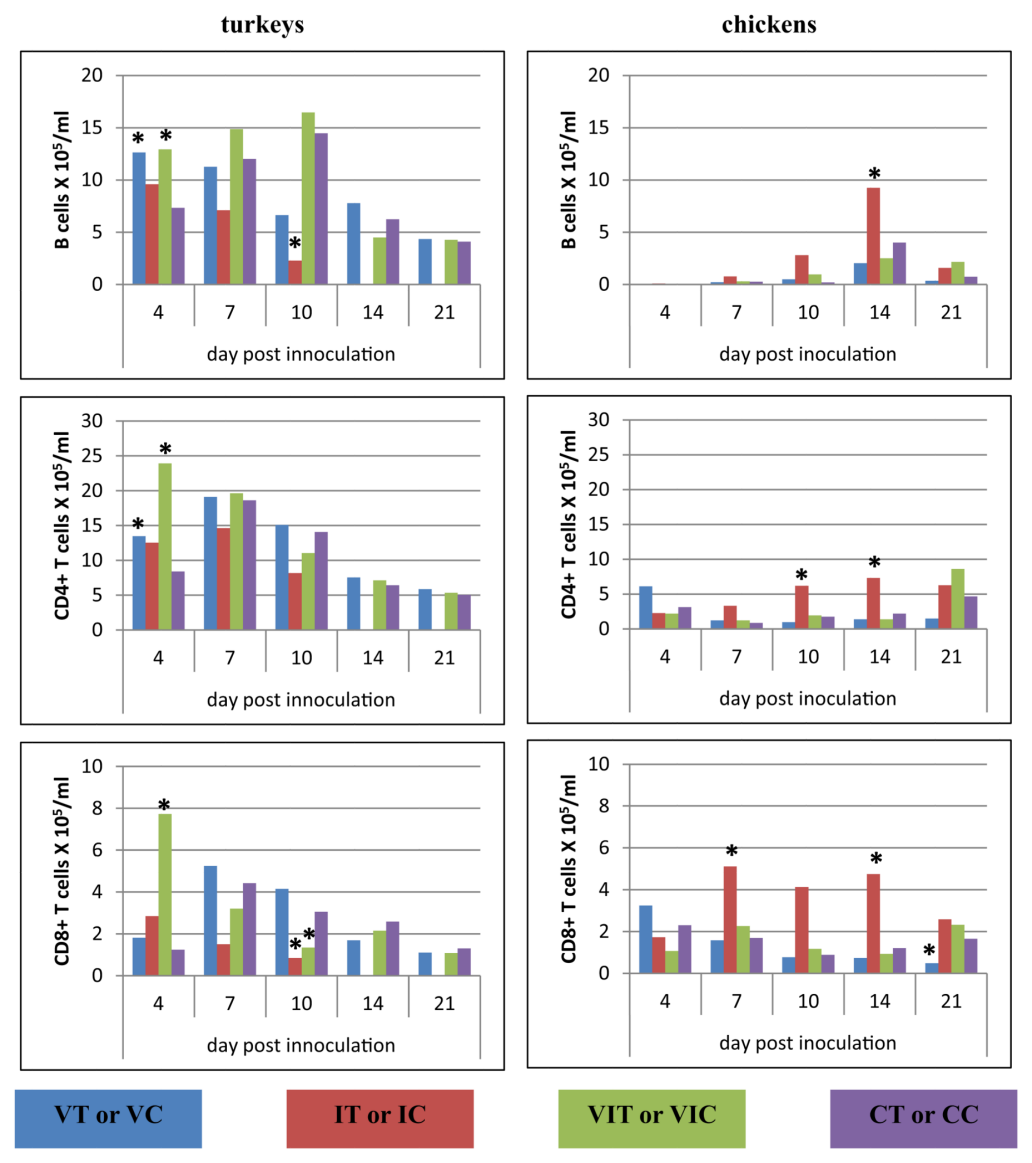
Alterations of B cells, CD4^+^ and CD8α^+^ T cells following vaccination and/or infection analysed by flow cytometry in the PBMCs. Absolute cell numbers of B cells (CD3ε^−^MHC-II^+^, turkey; CD3ε^−^Bu1^+^, chicken), CD4^+^ T cells (CD3ε^+^CD4^+^CD8α^−^) and CD8^+^ T cells (CD3ε^+^CD4^−^CD8α^+^) were determined in different groups of turkeys and chickens at different days post inoculation. Data represent the mean absolute cell number of 3 birds/group. *Significant different to control group of the respective species (P ≤ 0.05).

In PBMC (Fig. 7), CD4^+^ T and B cell populations significantly increased in groups VIT and VT together with CD8α^+^ T cells in group VIT at 4 DPI. B cells of chickens were detected in very low numbers in the blood on 4 DPI for all groups. Three days later CD8α^+^ T cells were increased in group IC. At 10 DPI group IT showed a decrease of B cells as well as CD8α^+^ T cells which were also found in lower amounts in birds of group VIT. Birds of group IC showed an increase of all lymphocytes at 14 DPI and on the last sampling day CD8α^+^ T cells were decreased in group VC. The groups VT and VIT did not show changes at 14 and 21 DPI.

#### 3.2.7 Total white blood cells of chickens

**Fig. 8:**
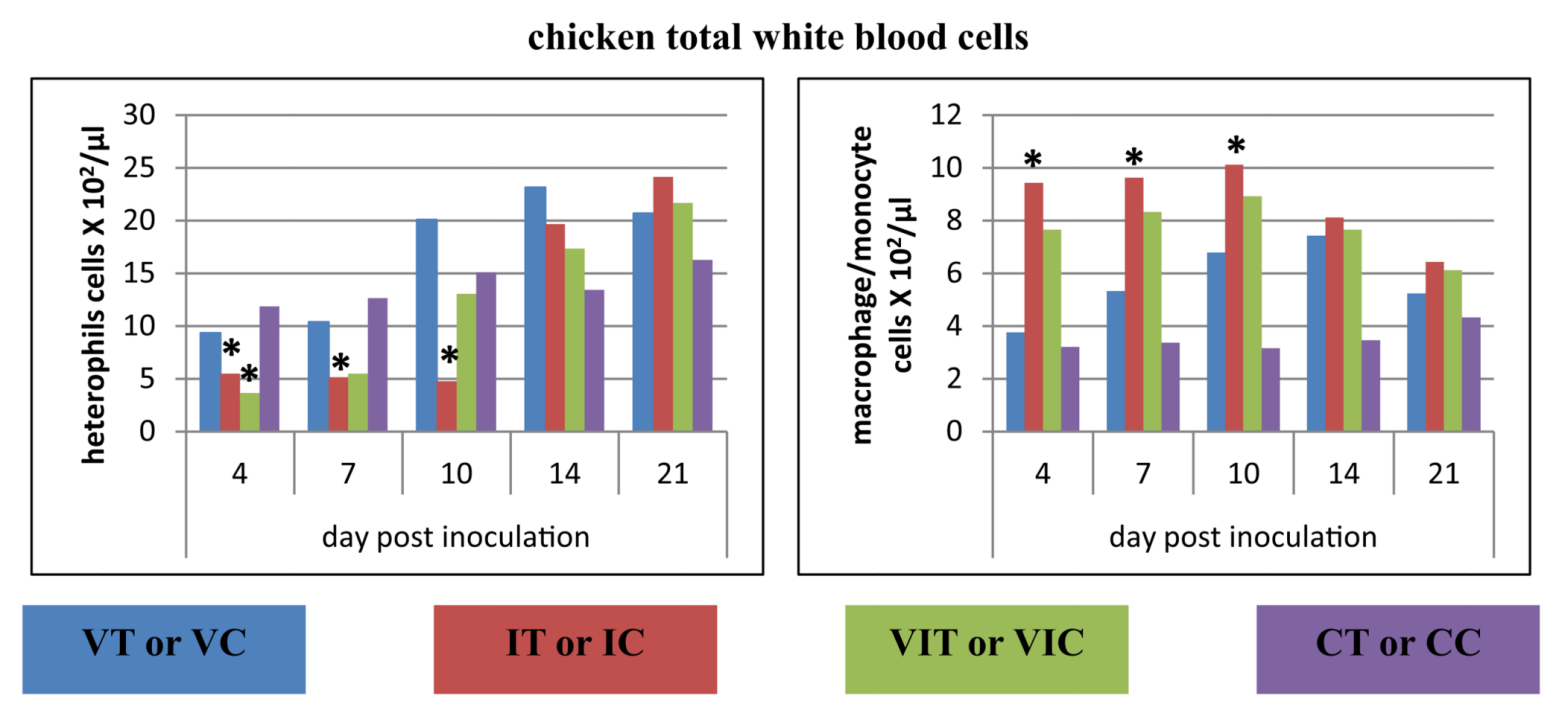
Analysis of total white blood cells in chickens. Absolute numbers of heterophils and monocytes/macrophages were calculated at different days post inoculation in whole blood of vaccinated chickens (VC), infected chickens (IC), vaccinated and infected chickens (VIC) together with control chickens (CC). Data represent the mean absolute cell number of the 3 birds/group. *Significant different to control group of the respective species (P ≤ 0.05).

The total white blood cell analysis was performed in order to address possible changes of absolute numbers of monocytes/macrophages and heterophils. Details of the absolute cell number for heterophils and macrophages/monocytes are given in Fig. 8. In group IC monocytes/macrophages significantly increased between 4 and 14 DPI, when heterophils were found to be significantly decreased. In group VIC a reduction of heterophils in the blood at 4 DPI was observed.

## 4 Discussion

The rising impact of histomonosis in flocks of turkeys and chickens without preventive or therapeutic options against the disease in industrial countries increases the importance and necessity to apply new strategies to combat the disease [3]. *In vitro* attenuated *H. meleagridis* was shown to reduce lesions and clinical signs in both bird species [7,9]. However, nothing is known on the immune response following vaccination. Furthermore, no data are available on changes of absolute numbers of immune cells following infection with virulent histomonads. Especially, a comparative study between turkeys and chickens suffering from histomonosis is of interest to elucidate cellular immune mechanisms that may contribute to the different clinical outcome of the disease in the two species.

In the present study, we focussed on major lymphocyte subsets representing cells of the adaptive immune system by investigating quantitative changes of B cells, CD4^+^ T cells and CD8α^+^ T cells in different organs and blood of birds inoculated with attenuated and/or virulent parasites. The applied attenuated and virulent histomonads were co-cultivated with bacteria which were initially isolated from birds suffering from histomonosis and characterized as species belonging to the normal gut microbiota [19]. In the last mentioned study, it was also reported that the virulence of the same clonal strain of *H. meleagridis* cultivated with a non-pathogenic laboratory strain of *E. coli* (DH5α) is independent of the co-cultivated bacteria. Anyhow, the bacterial flora of birds from all groups consisted of normal gut bacteria for a valid comparability.

As a pre-requisite it was crucial to identify appropriate mAbs for the detection of the aforementioned lymphocyte subsets in turkeys and chickens. For chickens, the respective antibodies are commercially available but there are no specific markers for turkey cells. The cross-reactivity of anti-chicken antibodies against B cells, CD4^+^ and CD8α^+^ of turkey was previously demonstrated [17,18,20,21], whereas anti-chicken CD3^+^ antibody failed to react with turkey T cells. In order to identify CD4^+^ and CD8α^+^ T cells, we tested the rat anti-human CD3-12 mAb (AbD serotec) for cross-reactivity against chicken and turkeys cells. The mAb recognizes a peptide representing an invariant cytoplasmic sequence within the CD3ε chain [22]. In several studies a broad species cross reactivity of this antibody was demonstrated in different mammals and birds like ducks and owls [22–28]. *In silico* analysis revealed a difference of only a single amino acid at the N-terminal end within the entire 14 amino acid sequence recognized by the antibody. Comparative FCM investigations using the species-specific mouse anti-chicken CD3 antibody in samples of chickens verified the validity of the rat anti-human CD3ε antibody to be used as a marker for chicken T cells. Similarly, co-stainings of the anti-human CD3ε antibody with established cross-reactive CD4 and CD8α-specific antibodies in turkey lymphocytes indicated that the antihuman CD3ε antibody CD3-12 can be used for the identification of turkey T cells.

In samples from chickens and turkeys that received vaccination, vaccination and infection, or only infection divergences in the numbers of B cells, CD3ε^+^CD4^+^CD8α^−^ and CD3ε^+^CD4^−^CD8α^+^ cells were observed by FCM: in the early stage of infection (4 DPI) the main changes were observed in vaccinated and infected turkeys (group VIT) when all lymphocyte subpopulations decreased in the caecum but increased in the blood. This may indicate a rapid apoptosis of effector memory cells in the caecum which are replenished by proliferating central memory cells via the blood stream. Similar to an early time point of infection, at 4 dpi, vaccination of naive turkeys rarely caused variations which might be explained by an unresponsiveness of the adaptive immune system at this early time point post inoculation. Lymphocytes of chickens, including vaccinated and infected birds, remained largely unchanged at 4 DPI. The milder response to the challenge of vaccinated chickens compared to turkeys might be interpreted as a much lower reactivation of *Histomonas*-specific memory cells after challenge at early stage compared to turkeys.

At 7 DPI CD4^+^ and CD8α^+^ T cells were depleted in the caecum of IT, resembling the pattern of T-cell subset changes in VIT at 4 DPI. In the liver all lymphocyte subpopulations of IT birds were increased and the same was observed for B cells and CD8α^+^ T cells of group VIT. This later response might be due to the delayed infiltration of the parasite into the liver compared to the caecum. In the spleen only CD4^+^ T cells increased in group IT as well as both T-cell subsets in group VIT, however, a more pronounced elevation of all cell types occurred in turkeys of group VT. This may indicate a systemic response in this group due to vaccination although this did not apply to PBMC. In chickens all cell populations were similar in number with some exceptions: most noticeable, the increase of B cells in caecum of birds in the VIC group may indicate a delayed recall response of B cells in this species compared to turkeys.

On day 10, the main cellular aberration was observed in group IT in the caecum where B cells and CD8α^+^ T cells were abundant but at the same time lower in number in the blood. This finding shows the composition of the investigated lymphocyte subsets shortly before turkeys succumb to histomonosis. Histomonosis causes severe inflammation and necrosis in caecum and liver of turkeys [2], this is confirmed in our study with highest lesion score for both the organs of infected turkeys on 10 DPI. This excessive necrosis may also result from the cytotoxic activity of CD8α^+^ T cells, acting as effector cells which was reported for the caecum of chickens following infections with the virulent protozoan parasite *Eimeria tenella* [29].

Interestingly, and in agreement with the aforementioned hypothesis infected chickens that did not contract the disease showed relatively unchanged cell numbers in the caecum. However, all lymphocytes increased (T-cell subsets significantly) in the liver. Compared to the turkey this response is again delayed and is in coherence with milder LS in the liver.

At the last two sampling days, 14 and 21 DPI, data from group IT was not available due to the fatal outcome of the infection. The remaining turkeys in group VT and VIT showed reduced numbers of all investigated lymphocytes in the caecum but increased amounts of CD4^+^ and CD8α^+^ cells in the liver, mostly with significant relevance. These cellular changes occurred at a later time point compared to birds from the IT group. Based on this observation, it might be concluded that the lymphocyte subsets of vaccinated turkeys showed a second wave of effector activity which results in increased lesion scores and is accompanied by a rapid death of these effector cells.

In chickens the analysed immune cells in infected organs were mostly in a normal range matching with the low LS. Mainly in the blood, an increase of B cells and both T cell subpopulations in group IC was observed at 14 DPI which may indicate the ongoing recruitment of these cells due to the infection with virulent histomonads.

Analyses of white blood cells from whole blood were performed to obtain quantitative data on macrophages/monocytes and heterophils but was restricted to samples from chickens due to the lack of markers for turkey cells [16]. Within the IC group macrophages/-monocytes resulted in significant higher amounts between 4 and 10 DPI compared to the control, suggesting the activation of an innate immune response which contributes to inactivate the parasite. The heterophils were significantly lower during that time in the blood this may be due to the infiltration of these granulocytes in the local site of infection. The severe infiltration of mononuclear and polymorphonuclear cells in the infiltrated organs caecum and liver is a common histopathological finding in the course of histomonosis [30].

Overall, quantitative analyses of the present study suggest that CD4^+^ and CD8α^+^ T cells are involved in the immune response against histomonosis which is coherent with previous results based on cytokine expression and histological assays [11]. Furthermore, it was found in the actual study that attenuated histomonads caused a much lower variation of the T-cell subsets and B cells, often equal to the control group. This reduced cellular response could also be noticed after infection of vaccinated birds with virulent histomonads. Moreover, it could be demonstrated that the fatal clinical outcome of turkeys due to histomonosis is in coherence with a more intense cellular immune response in infected organs compared to chickens.

## Figures and Tables

**Table 1:** Experimental vaccination and/or infection of chickens and turkeys. Post mortem examinations and sampling of organs together with blood from 3 birds per group for flow cytometry experiments were performed on 4, 7, 10, 14 and 21 days post inoculation.

Group	Day of life/day post inoculation						
	
	1	28	32/4	35/7	38/10	42/14	49/21
Vaccinated turkeys (VT)		Vaccination	x^a^	x	x	x	x
Vaccinated chickens (VC)			x	x	x	x	x
Infected turkeys (IT)		Infection	x	x	x	n.a.^b^	n.a.
Infected chickens (IC)			x	x	x	x	x
Vaccinated and infected turkeys (VIT)	Vaccination	Infection	x	x	x	x	x
Vaccinated and infected chickens (VIC)			x	x	x	x	x
Control turkeys (CT)			x	x	x	x	x
Control chickens (CC)			x	x	x	x	x

a Necropsy and sampling of three birds.

b Not applicable due to fatal histomonosis at earlier time points.

**Table 2:** Antibody panels. List of antibodies and antibody combinations used in this study.

Purpose	Species specificity	Antigen	Clone	Isotype	Fluoro-chrome	Labeling strategy	Source of primary mAb
Cross-reactivity of CD3-12	Human	CD3ε	CD3-12	Rat IgG1	AlexaFluor 647	Directly conjugated	AbD Serotec
	Chicken	CD3	CT3	Mouse IgG1	BV421	Biotin-streptavidin^a^	Southern-Biotech

*H. meleagridis* vaccination/infection study: Turkey panel 1	Human	CD3ε	CD3-12	Rat IgG1	AlexaFluor 647	Directly conjugated	AbD Serotec
	Chicken	MHC-II	2G11	Mouse IgG1	BV421	Secondary antibody^b^	LMU Munich^d^

Turkey panel 2	Human	CD3ε	CD3-12	Rat IgG1	AlexaFluor 647	Directly conjugated	AbD Serotec
	Chicken	CD4	CT4	Mouse IgG1	BV421	Biotin-streptavidin^a^	Southern-Biotech
	Chicken	CD8α	3-298	Mouse IgG2b	R-PE	Directly conjugated	Southern-Biotech

Chicken panel 1	Human	CD3ε	CD3-12	Rat IgG1	AlexaFluor 647	Directly conjugated	AbD Serotec
	Chicken	Bu-1	AV20	Mouse IgG1	BV421	Biotin-streptavidin^a^	Southern-Biotech

Chicken panel 2	Human	CD3ε	CD3-12	Rat IgG1	AlexaFluor 647	Directly conjugated	AbD Serotec
	Chicken	CD4	CT4	Mouse IgG1	BV421	Biotin-streptavidin^a^	Southern-Biotech
	Chicken	CD8α	3-298	Mouse IgG2b	R-PE	Directly conjugated	Southern-Biotech

Chicken whole blood panel	Chicken	CD45	16-4	Mouse IgG2a	PerCP	Conjugation kit^c^	Southern-Biotech
	Chicken	Bu-1	AV20	Mouse IgG1	APC	Conjugation kit^c^	Southern-Biotech
	Chicken	Macrophags/monocytes	Kul-01	Mouse IgG1	R-PE	Conjugation kit^c^	Southern-Biotech
	Chicken	CD4	CT4	Mouse IgG1	FITC	Directly conjugated	Southern-Biotech
	Chicken	CD8α	3-298	Mouse IgG2b	FITC	Directly conjugated	Southern-Biotech
	Chicken	TCR-γδ	TCR1	Mouse IgG1	FITC	Directly conjugated	Southern-Biotech

a Brilliant Violet 421™ Streptavidin, BioLegend.

b Rat anti-mouse IgG1 Brilliant Violet 421™, clone RMG1-1, BioLegend.

c LYNX Rapid Conjugation kits^®^, AbD serotec.

d Kindly provided by Prof. Dr. Thomas Göbel; Institute of Animal Physiology, Ludwig-Maximilian University, Munich, Germany.

**Table 3:** Amino acid sequence alignment of the epitope recognized by the monoclonal antibody CD3ε (clone CD3-12). Comparison of the relevant amino acid sequence of human T-cell surface glycoprotein CD3ε chain with the respective CD3ε molecule of chicken and turkey using protein BLAST^®^. The difference in the amino acid sequence is denoted in red colour.

Description	Sequence	Identity	Accession No.
CD3 epsilon chain precursor (*Homo sapiens*)	ERPPPVPNPDYEPI	100%	NP 000724.1
CD3 epsilon chain isoform X1(*Gallus gallus*)	QRPPPVPNPDYEPI	92%	XP 015153448.1
CD3 epsilon chain (*Gallus gallus*)	QRPPPVPNPDYEPI	92%	ACF04800.1
CD3 epsilon chain precursor (*Gallus gallus*)	QRPPPVPNPDYEPI	92%	NP 996787.1
CD3 epsilon chain (*Meleagris gallopavo*)	QRPPPVPNPDYEPI	92%	XP 003212775.1

## Abbreviations

DPI: day post inoculation
FCM: flow cytometry
VT: vaccinated turkeys
VC: vaccinated chickens
IT: infected turkeys
IC: infected chickens
VIT: vaccinated and infected turkeys
VIC: vaccinated and infected chickens
CT: control turkeys
CC: control chickens
LS: lesion score
PBS: phosphate buffered saline
FCS: fetal calf serum
PBMCs: peripheral blood mononuclear cells
IELs: intraepithelial lymphocytes
aa: amino acid
mAb: monoclonal antibody
